# Quantifying the Measurement Precision of a Commercial Ultrasonic Real-Time Location System for Camera Pose Estimation in Indoor Photogrammetry

**DOI:** 10.3390/s26010319

**Published:** 2026-01-03

**Authors:** Faith Nayko, Derek D. Lichti

**Affiliations:** Department of Geomatics Engineering, The University of Calgary, 2500 University Drive NW, Calgary, AB T2N 1N4, Canada; ddlichti@ucalgary.ca

**Keywords:** bundle adjustment, camera pose estimation, direct georeferencing, indoor positioning system, integrated sensor orientation, photogrammetry, real-time location system, ultrasonic positioning

## Abstract

Photogrammetric reconstruction from indoor imagery requires either labor-intensive ground control points (GCPs) or positioning sensor integration. While global navigation satellite system technology revolutionized aerial photogrammetry by enabling direct georeferencing through integrated sensor orientation (ISO), indoor environments lack an equivalent positioning solution. Before indoor positioning systems can be adopted for photogrammetric applications, their fundamental measurement precision must be established. This study characterizes the repeatability and temporal stability of the ZeroKey Quantum real-time location system (RTLS) as a prerequisite to testing reconstruction accuracy when RTLS measurements provide camera pose constraints in photogrammetric bundle adjustment. Through systematic tripod-mounted observations across 30 test locations in a controlled laboratory environment, optimal data collection protocols were determined, temporal stability was investigated, and measurement precision was quantified. An automated position-based stationary detection algorithm using a 20 mm threshold successfully identified all 30 stationary periods for durations of 30 s or less. Optimal duration analysis revealed that 1 s observation windows achieve 3 mm position precision and 1° orientation precision after brief settling, enabling practical workflows with worst-case total collection time of 2.5 s per station. Per-axis uncertainties were quantified as 1.6 mm, 1.7 mm, and 1.1 mm root mean square (RMS) for position and 0.08°, 0.09°, and 0.07° RMS for orientation. These findings demonstrate that ultrasonic RTLS achieves millimeter-level position repeatability and sub-degree orientation repeatability, establishing the measurement precision necessary to justify subsequent accuracy testing through photogrammetric bundle adjustment.

## 1. Introduction

### 1.1. Background

Photogrammetric reconstruction produces three-dimensional (3D) models from overlapping photographs, but without external reference information, these models exist in an arbitrary coordinate system with unknown scale. Resolving the scale ambiguity is essential for extracting metric information from the reconstruction. When aerial photogrammetry was first developed, this was achieved through extensive networks of ground control points (GCPs)—points with known coordinates that tie the reconstructed model to a global coordinate system and established correct scale. However, establishing and surveying these GCP networks was labor-intensive, severely limiting operational efficiency. The integration of global navigation satellite system (GNSS) receivers with aerial cameras in the 1990s revolutionized the field by enabling direct georeferencing through integrated sensor orientation (ISO), where GNSS measurements are incorporated as weighted observations in photogrammetric bundle adjustment [1,2]. GNSS-assisted aerial triangulation dramatically reduced ground control requirements, and combined GNSS/inertial navigation system solutions enabled high-accuracy reconstruction with minimal or no GCPs [3,4]. This technological advancement transformed aerial photogrammetry from a ground-control-intensive process into a rapid, efficient workflow.

Indoor environments lack an equivalent positioning solution. GNSS signals do not penetrate building structures, rendering GNSS-based ISO impossible for indoor applications. This limitation motivates investigation of indoor positioning systems (IPSs) that could provide camera pose constraints for indoor photogrammetric reconstruction.

### 1.2. Related Work

IPSs have been implemented using various positioning technologies, including Wi-Fi, Bluetooth Low Energy (BLE), ultra-wideband (UWB), and ultrasonic technologies. Comprehensive surveys have examined these technologies from perspectives of accuracy, coverage, cost, and application suitability [5,6,7]. Wi-Fi fingerprinting typically achieves meter-level accuracy [5], while BLE fingerprinting achieves 1–3 m accuracy [6]. UWB systems achieve 10–30 cm accuracy through time-of-arrival ranging [8], and ultrasonic ranging achieves 1–50 cm accuracy, with some implementations reaching millimeter-level performance [9,10]. Of these technologies, only UWB and ultrasonic technologies demonstrate accuracy potentially suitable for photogrammetric applications.

Significant research has addressed the integration of positioning systems with visual systems for dynamic applications involving moving cameras. While UWB-vision fusion has been investigated extensively for real-time applications [11,12,13,14,15], ultrasonic-vision integration has received comparatively less attention [16]. These approaches employ sensor fusion frameworks—such as visual odometry [11], visual-inertial odometry [13], and simultaneous localization and mapping [14]—that continuously estimate position as the camera moves. Such frameworks prioritize real-time responsiveness and trajectory continuity for applications such as mobile robotics, pedestrian navigation, and unmanned aerial vehicle (UAV) localization.

These sensor fusion frameworks can be characterized as tightly or loosely coupled. Tightly coupled integration techniques fuse raw measurements from multiple sensors simultaneously within a single estimation framework [14,17]. Loosely coupled techniques instead combine already-processed outputs from independent systems [18]. The present study proposes a loosely coupled approach where RTLS position and orientation measurements serve as weighted constraints in photogrammetric bundle adjustment. Regardless of fusion approach, calibration for sensor error models may be required depending on system characteristics [19], and geometric calibration of mounting parameters (lever arm and boresight) is necessary for accurate direct georeferencing [20,21,22]. However, mounting parameter calibration is beyond the scope of this precision characterization study.

For photogrammetric applications specifically, existing positioning integration approaches have employed post-processing methods rather than direct incorporation as weighted constraints within bundle adjustment. Reference [23] demonstrated UWB-based positioning for UAV photogrammetry by applying a post-processing transformation to align reconstruction with the positioning coordinate frame, while [24] used ultrasonic positioning for post-processing depth correction of structure-from-motion point clouds. Post-processing approaches can only correct the final reconstruction as a rigid body, treating systematic errors uniformly across the entire model. In contrast, ISO incorporates positioning measurements directly into bundle adjustment optimization, allowing the solution to account for measurement uncertainties at each camera station.

In summary, existing research on positioning-vision integration exhibits three key gaps: (1) UWB has received substantially more attention than ultrasonic positioning, despite ultrasonic systems being capable of superior accuracy; (2) integration approaches have focused on real-time tracking for dynamic applications rather than precise pose estimation at discrete capture instants; and (3) photogrammetric applications have employed post-processing corrections rather than direct incorporation of positioning measurements as weighted constraints in bundle adjustment. Systematic characterization of ultrasonic positioning system precision for stationary camera pose estimation, as required for photogrammetric ISO, remains relatively unexplored.

### 1.3. Research Objectives

These gaps motivate evaluation of a real-time location system (RTLS) as a positioning solution for indoor photogrammetry. The present study quantifies the measurement precision of the Quantum RTLS (ZeroKey Inc., Calgary, AB, Canada)—a commercial ultrasonic positioning system with an integrated inertial measurement unit (IMU) and manufacturer-specified position accuracy of 1.5 mm [25]. The envisioned operational workflow involves efficient handheld data collection where an operator walks through a space, pausing briefly at each station to capture imagery and record pose estimates—eliminating both GCPs and tripod setup. Realizing this workflow requires a positioning system with sufficient measurement accuracy. However, precision is a necessary prerequisite for accuracy: sensors with poor repeatability cannot provide accurate constraints. This study establishes precision thresholds appropriate for photogrammetric applications and identifies data collection protocols necessary for the RTLS to achieve them.

Before the envisioned handheld workflow can be validated, fundamental RTLS measurement characteristics must be established under controlled conditions. This study therefore employs tripod-mounted observations to establish optimal data collection protocols and quantify achievable precision. This approach isolates RTLS measurement behavior from operator motion artifacts, providing the empirical foundation required before progressing to handheld deployment.

This paper addresses the following research objectives in a controlled laboratory environment:

1. Develop an automated stationary detection algorithm to support the envisioned walk-around workflow, enabling the system to detect when the operator has paused and signal when measurements meeting precision thresholds have been acquired.
2. Evaluate IMU sensor fusion performance across all five proprietary configuration profiles to identify which provides suitable orientation constraints for stationary photogrammetric observations.
3. Determine optimal data collection protocols by evaluating both duration (observation windows from 0.25 s to 90 s) and strategy (immediate collection versus threshold-based settling).
4. Quantify achievable position and orientation precision under stationary conditions, establishing baseline performance characteristics to inform the weighting of camera pose constraints in photogrammetric bundle adjustment.

The findings provide the empirical foundation necessary for subsequent accuracy testing of RTLS-assisted photogrammetric reconstruction, establishing whether ultrasonic positioning can offer indoor photogrammetry what GNSS provided for aerial photogrammetry—a practical alternative to ground control.

### 1.4. Organization

The remainder of this paper is organized as follows: Section 2 describes the ZeroKey Quantum RTLS architecture, experimental design, and analytical framework. Section 3 presents experimental results and analysis, including stationary detection algorithm evaluation, IMU profile comparison, optimal data collection protocol analysis, and precision quantification. Section 4 synthesizes the findings and establishes accuracy testing as the focus for subsequent work.

## 2. Materials and Methods

### 2.1. ZeroKey Quantum RTLS

The ZeroKey Quantum RTLS is a commercial ultrasonic IPS. The following system description is based on the manufacturer’s white paper [26] and technical documentation [27].

**Figure 1:**
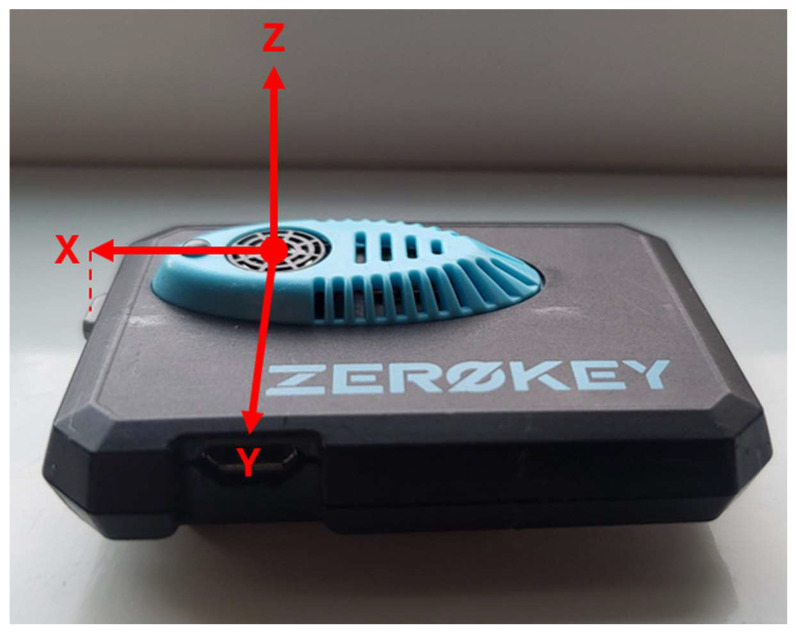
ZeroKey mobile node body frame coordinate system. The origin is located at the center of the ultrasonic transducer, with the positive X-axis pointing toward the action button, the positive Y-axis pointing toward the micro-USB port, and the Z-axis completing a right-handed coordinate system. Adapted from [28].

The system comprises fixed anchor nodes with known positions, one or more mobile nodes that serve as the tracked devices, and a Gateway that serves as the communications bridge to a connected computer. The mobile node body frame coordinate system is defined with its origin at the center of the ultrasonic transducer, as shown in Figure 1. The positive X-axis points toward the device’s action button, the positive Y-axis points toward the micro-USB port, and the Z-axis completes a right-handed coordinate system.

The Quantum RTLS implements a hybrid electromagnetic-ultrasonic ranging protocol that eliminates the strict clock synchronization requirements of conventional ToF systems. When the mobile device initiates a ranging sequence, it transmits both an electromagnetic signal and an ultrasonic pulse simultaneously. The electromagnetic wave arrives at the anchor node essentially instantaneously and serves as a precise time reference, while the ultrasonic pulse arrives after a measurable delay. By comparing the arrival times of these two signals, the anchor node can calculate the distance to the mobile node without requiring synchronized clocks between devices.

The system fuses range measurements from multiple anchors with inertial measurements from the mobile node’s integrated IMU to produce six-degree-of-freedom pose estimates. The position of the mobile node transducer is expressed as Cartesian coordinates (X, Y, Z), and the orientation of the mobile node body frame relative to the local anchor network coordinate system is expressed as a unit quaternion.

The system requires the mobile device to have line-of-sight to a minimum of four anchors at all times for 3D positioning [29]. Before operation, the anchor network must undergo a guided calibration procedure to establish the local coordinate system [30]. The local frame is a right-handed Cartesian coordinate system with the negative Z-axis aligned with local gravity. The system selects an arbitrary anchor as the origin. Heading is also defined arbitrarily. The proprietary nature of the calibration algorithm prevents direct examination of how anchor geometry influences coordinate system establishment.

The integrated IMU provides orientation estimates through five selectable profiles optimized for different motion patterns [31]: Profile 1 (Pedestrian), Profile 2 (Tooltip), Profile 3 (Hybrid Autonomous Guided Vehicle), Profile 4 (Autonomous Guided Vehicle), and Profile 5 (Generic). Each profile employs proprietary sensor fusion algorithms and requires an initialization training procedure each time positioning begins to train the sensor fusion algorithm for the expected motion characteristics. All five profiles were evaluated empirically to determine which demonstrates precision characteristics best suited for photogrammetric bundle adjustment.

### 2.2. Experimental Design and Data Collection

#### 2.2.1. Test Environment and Infrastructure

Experiments were conducted in the Geospatial Vision Metrology Laboratory at the University of Calgary, where a ZeroKey Quantum RTLS was deployed to provide positioning coverage over a 6 m × 8 m floor area. Eight anchor nodes were mounted on the walls and ceiling at heights ranging from 2.3 m to 3.2 m above the floor, selected to provide optimal geometric coverage at the typical handheld camera height of approximately 1.5 m, reflecting the intended smartphone photogrammetric workflow. The anchor network geometry was designed to satisfy three requirements:

1. Ensuring at least four anchors maintain line-of-sight to any position within the tracked volume to enable robust positioning.
2. Maximizing geometric diversity through varied anchor heights and spatial distribution to strengthen positioning accuracy throughout the test area.
3. Maintaining practical installation constraints by mounting anchors on existing walls and ceiling surfaces without specialized infrastructure.

**Figure 2:**
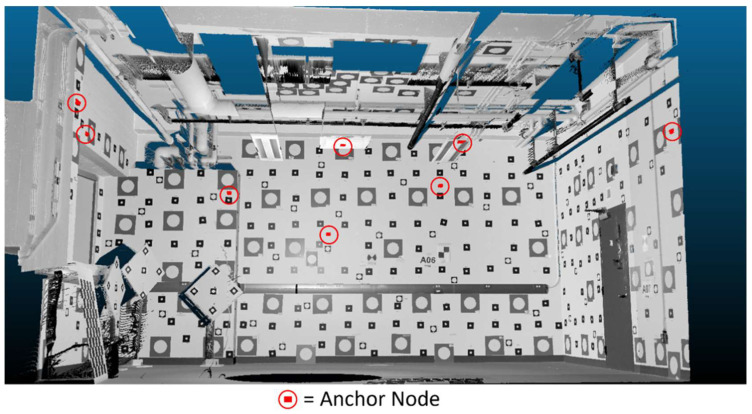
Perspective view of the laboratory test environment showing anchor node deployment. Eight anchor nodes are distributed around the perimeter and overhead of the 6 m × 8 m test area, visualized using a high-resolution terrestrial laser scanning point cloud.

Figure 2 presents a perspective view of the test area (rendered from a point cloud acquired via terrestrial laser scanning) showing the eight anchor node positions within the laboratory space. The anchor network was calibrated using the manufacturer’s guided calibration procedure to establish the local coordinate system.

**Figure 3:**
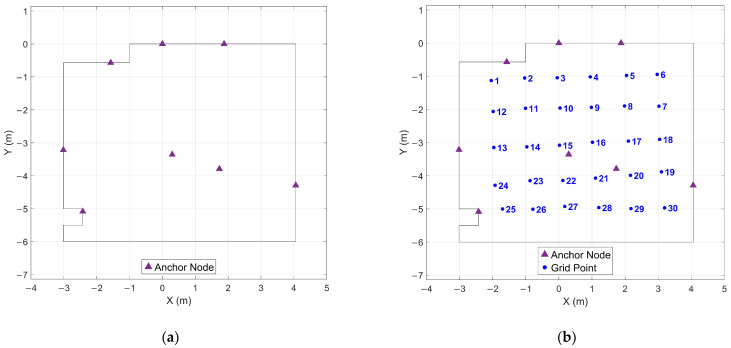
Anchor network geometry and test grid layout in plan view: (**a**) Eight anchor nodes distributed around the perimeter of the 6 m × 8 m floor area; (**b**) Anchor network with regular grid of 30 test points at 1 m spacing covering the central region.

Figure 3 shows the anchor network geometry in plan view, illustrating the 6 m × 8 m floor area and the distribution of anchor nodes around the perimeter and overhead. A regular grid of 30 test points with 1 m spacing was established within this area, covering the central region where geometric constraints from the anchor network are strongest. These grid points served as observation locations for systematic data collection, enabling evaluation of positioning performance as a function of location within the tracked volume.

#### 2.2.2. Data Collection

Data collection followed a systematic protocol designed to evaluate measurement repeatability and temporal stability. At each grid point, a ZeroKey mobile node was positioned on a tripod with the transducer facing upward at approximately 1.5 m height—approximating typical handheld camera positions—and remained stationary for 90 s while continuously logging position and orientation estimates at 20 Hz. This tripod-mounted approach isolated RTLS measurement characteristics from operator motion artifacts, establishing proof-of-concept validation under controlled conditions before progressing to handheld deployment.

The 90 s observation duration was selected to enable investigation of temporal stability and convergence characteristics while remaining practical for multi-station surveys. Each stationary period provided approximately 1800 pose estimates (90 s × 20 Hz), enabling robust statistical characterization of measurement precision and systematic behavior.

Data collection was repeated across all 30 grid points for each of the five IMU profiles, generating comprehensive datasets for profile comparison. Prior to data collection for each profile, the system was initialized according to the manufacturer’s instructions.

### 2.3. Analytical Framework

#### 2.3.1. Stationary Period Detection

Automated detection of stationary periods from the continuous data stream is essential for the envisioned walk-around workflow. A position-based detection approach using consecutive position differences was implemented, avoiding potential issues with velocity estimates derived from internal filtering rather than direct measurement. The algorithm computed the Euclidean distance between consecutive position estimates at the 20 Hz sampling rate, classifying samples as stationary when the distance fell below a specified threshold. Stationary periods were then identified as continuous runs of stationary samples exceeding a minimum duration requirement.

The position threshold parameter required empirical determination. To identify the minimum threshold achieving reliable stationary detection, systematic testing was performed across threshold values of 15 mm, 20 mm, and 25 mm. For each threshold, detection success was assessed by comparing the number of detected periods against the 30 known stationary periods, with ground truth boundaries established by timestamps recorded during data collection. The interaction between threshold and minimum duration was also characterized by varying the minimum duration parameter from 1 s to 25 s, with particular attention to whether detected period durations matched the true stationary period lengths (nominally 90 s).

Following threshold determination, the optimal threshold was adopted for all subsequent analyses. Detection performance was evaluated across the five IMU profiles and three stationary duration conditions (30 s, 60 s, and 90 s), measuring both the number of periods successfully identified and the 3D precision achieved for each detected period.

#### 2.3.2. Position and Orientation Estimate Analysis

This analysis used the full 90 s collections at each grid point to establish baseline precision metrics and identify any temporal or spatial performance patterns that would influence subsequent data collection optimization.

For orientation analysis, quaternions from each 90 s period were averaged using the Markley method [32] to ensure mathematically rigorous averaging across the rotation space. For photogrammetric applications, the orientation quaternions were converted to Cardan angles (ωϕκ), representing sequential rotations about the X, Y’, and Z’’ axes and aligning with standard close-range photogrammetric notation for camera pose parameters [33].

The tripod-mounted data collection employed the mobile node with the ultrasonic transducer facing upward, simulating the envisioned smartphone mounting configuration where the mobile node would be rigidly attached to a smartphone held in landscape orientation and tilted upward to capture imagery. This mounting orientation results in nominal camera orientations of ω ≈ 90° (depending on the upward tilt angle), ϕ ≈ 0° (representing the approximate heading at which the mobile node was manually placed), and κ ≈ 0° (due to the landscape orientation). This physically realistic configuration enabled the measured orientations to be assessed against known nominal angles to validate whether the RTLS produces accurate orientation measurements for the intended photogrammetric application.

Position precision was characterized by 95% confidence ellipses for horizontal (XY) components and 95% confidence intervals for vertical (Z) components. Standard deviations were calculated separately for each Cardan angle to quantify rotational stability and identify component-specific characteristics.

#### 2.3.3. Optimal Duration Analysis

Optimal duration analysis employed a two-stage approach to determine the minimum observation time required at each station to achieve pose estimates meeting precision thresholds. Candidate durations ranging from 0.25 s to 90 s were systematically evaluated, with the minimum viable duration set to 0.25 s (5 epochs at 20 Hz) to ensure reliable precision calculations.

The 3D position precision threshold was set at 3 mm—double the manufacturer’s 1.5 mm accuracy specification—to account for potential systematic errors and ensure robust performance under varying conditions. The precision threshold for each Cardan angle was set at 1°, a conservative value that the system easily satisfied. At the achieved precision level of approximately 0.1° (Section 3.4), orientation uncertainty contributes less than 9 mm positional uncertainty at object distances of 5 m, typical of indoor close-range applications. 

First-window analysis evaluated the precision achievable from immediate data collection by extracting the initial portion of each 90 s dataset for each candidate duration and computing the resulting position and orientation precision. For each candidate duration, the mean and range of 3D position precision and per-angle orientation precision values across all 30 grid points were computed to characterize both typical performance and variability between locations.

Building on these baseline results, sliding-window threshold analysis identified the earliest window within each stationary period that achieved the precision criterion. Position and orientation measurements were analyzed independently, as their precision may evolve differently over time. Windows of fixed duration were advanced through the 90 s stationary period at 0.05 s (1-epoch) intervals. The analysis recorded:

1. Success rate: the percentage of grid points where the precision criterion was met.
2. Wait time: the delay from the start of the stationary period until the start of the first threshold-meeting window.
3. Collection time: wait time plus window duration.
4. Final precision: the precision values achieved during the first threshold-meeting window.

An optimal collection duration was selected based on these results—the shortest duration achieving 100% success rate for both position and orientation. Visualizations of the 95% position confidence regions were prepared for this optimal duration using both the first-window and sliding-window threshold approaches, demonstrating the improvements achievable through threshold-based settling compared to immediate data collection.

#### 2.3.4. Observation Uncertainty Quantification

Lastly, per-component position and orientation precision were quantified to inform the weighting of camera pose constraints in photogrammetric bundle adjustment. Using the earliest windows meeting both position and orientation thresholds at the optimal duration across all grid points, individual standard deviations for each position axis and each Cardan angle were computed, along with their root mean square (RMS) values. This approach provides a balanced characterization of measurement uncertainty that accounts for spatial variability without being dominated by outliers.

## 3. Results

### 3.1. Stationary Period Detection

#### 3.1.1. Threshold Determination

Systematic testing across threshold values of 15 mm, 20 mm, and 25 mm identified 20 mm as the minimum threshold achieving reliable detection of all 30 stationary periods.

Thresholds below 20 mm resulted in over-detection due to fragmentation of legitimate stationary periods. At 15 mm with 1 s minimum duration, 32 periods were detected instead of 30, with station 6—as shown in Figure 3b—fragmented into three separate detections (67.3 s, 6.7 s, and 20.4 s) due to instantaneous position jitter exceeding the stricter threshold. This fragmentation was consistent across minimum durations from 1 s to 10 s. Increasing the minimum duration to 25 s filtered the spurious fragments (yielding 30 detections), but the detected duration for station 6 remained only 67.3 s compared to the nominal 90 s stationary period. Correct detection of the full stationary period would require minimum durations exceeding 67 s, contradicting the goal of rapid data collection.

Conversely, increasing the threshold to 25 mm introduced a minor false positive at 1 s minimum duration: 31 periods were detected, including a spurious 1.1 s detection during a transition between stations. This was easily resolved by increasing the minimum duration to 5 s, which achieved correct detection of all 30 periods with accurate durations. Unlike the 15 mm threshold, which required impractically long minimum durations to avoid fragmenting legitimate stationary periods, the 25 mm threshold remained viable for efficient workflows. This asymmetry suggests that erring toward larger thresholds is preferable, as spurious transition detections are more easily filtered than fragmented stationary periods.

The 20 mm threshold achieved 100% detection success at minimum durations as short as 1 s, accommodating instantaneous sample-to-sample variability while maintaining sensitivity to true stationary periods. While this threshold exceeds the manufacturer’s 1.5 mm accuracy specification by an order of magnitude, it reflects instantaneous jitter characteristics of the real-time sensor fusion algorithm rather than time-averaged precision. The precision analysis in Section 3.4 confirms that millimeter-level repeatability is achieved once stationarity is established and measurements are averaged over appropriate collection windows. The 20 mm threshold was adopted for all subsequent stationary detection analyses.

#### 3.1.2. Detection Performance

The automated stationary detection algorithm was evaluated across the five IMU profiles at three stationary period durations (30 s, 60 s, and 90 s) to assess detection reliability and characterize precision across profiles. Table 1 presents detection results showing the number of successfully detected periods, detection accuracy percentages, undetected point IDs, and 3D precision statistics for each profile-duration combination.

All five IMU profiles achieved 100% detection of 30 stationary periods at 30 s duration, with mean 3D precision ranging from 2.4 mm (Profile 2) to 4.1 mm (Profile 5). However, extending stationary periods to 60 s and 90 s reduced detection reliability for Profiles 1, 4, and 5, while Profiles 2 and 3 maintained 100% detection across all durations. Profile 1 showed the most severe duration sensitivity, declining to 90% for 90 s periods (failures at grid points 1, 22, and 26). Profiles 4 and 5 exhibited moderate duration sensitivity, with detection rates dropping to 97% at 60 s and further declining at 90 s. These findings indicate that RTLS’s proprietary filtering algorithms prioritize real-time responsiveness over extended stationary stability, making longer collection periods counterproductive for certain profiles.

Profile 2 was selected for all subsequent analyses due to its consistent 100% detection performance across all durations and its superior precision characteristics—both the best mean precision (2.4 mm) and the best maximum precision (5.9 mm) among the five profiles. While Profile 3 showed similar detection performance, Profile 2′s precision and design characteristics make it the optimal choice for the envisioned workflow. All subsequent results present data from Profile 2 only, with complete results for all five profiles available in the Appendix A to support application-specific optimization.

### 3.2. Position and Orientation Estimate Analysis

**Figure 4:**
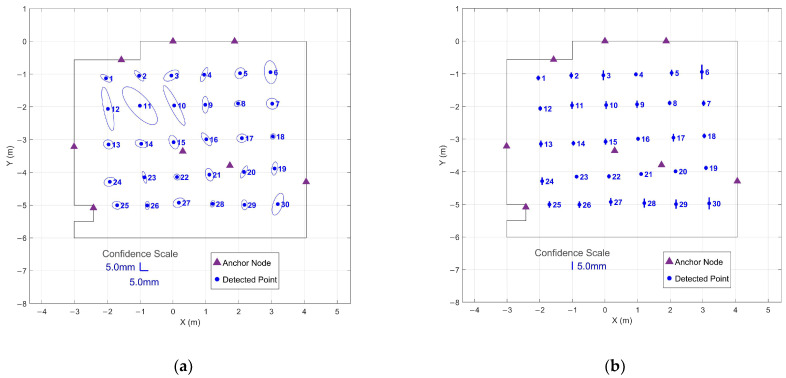
95% confidence regions from 90 s stationary periods at all 30 grid points: (**a**) Horizontal confidence ellipses showing XY precision across the tracked area; (**b**) Vertical confidence intervals showing Z precision for each grid point.

Position estimates demonstrated consistent sub-centimeter precision across the tracked volume. Figure 4 presents the 95% confidence regions calculated from the 90 s stationary periods at all 30 grid points. Horizontal confidence ellipses achieved a mean semi-major axis length of 5.1 mm with a maximum of 15.3 mm, while vertical confidence intervals averaged 2.0 mm with a maximum of 4.5 mm.

Orientation estimates demonstrated excellent temporal precision but exhibited expected spatial variation. Table 2 presents orientation statistics from 90 s stationary periods across all 30 grid points, showing both spatial mean values (variation across grid points) and temporal precision (mean standard deviation within each 90 s period).

Estimates for ω clustered tightly around 89.6°, demonstrating excellent agreement with the nominal 90° pitch angle for the tilted smartphone configuration. Measurements for κ centered at −0.4°, closely matching the expected 0° roll angle for landscape orientation. Values for ϕ ranged from −22.8° to 10.6°, with a spatial standard deviation of 8.3°—substantially larger variation compared to ω and κ. This variation reflects physical heading differences resulting from manual rig placement at each grid point rather than measurement instability, as visual alignment without indexed mounting hardware introduces heading variability.

Temporal standard deviations, averaged across all 30 grid points, demonstrated excellent measurement stability at individual locations, with ω achieving 0.07° ± 0.05°, ϕ achieving 0.12° ± 0.09°, and κ achieving 0.08° ± 0.07°. The temporal precision of ϕ—two orders of magnitude better than its spatial variation—confirms that the system accurately tracks heading changes, with observed spatial variation reflecting real differences in placement rather than measurement instability.

### 3.3. Optimal Duration Analysis

#### 3.3.1. First-Window Analysis

**Figure 5:**
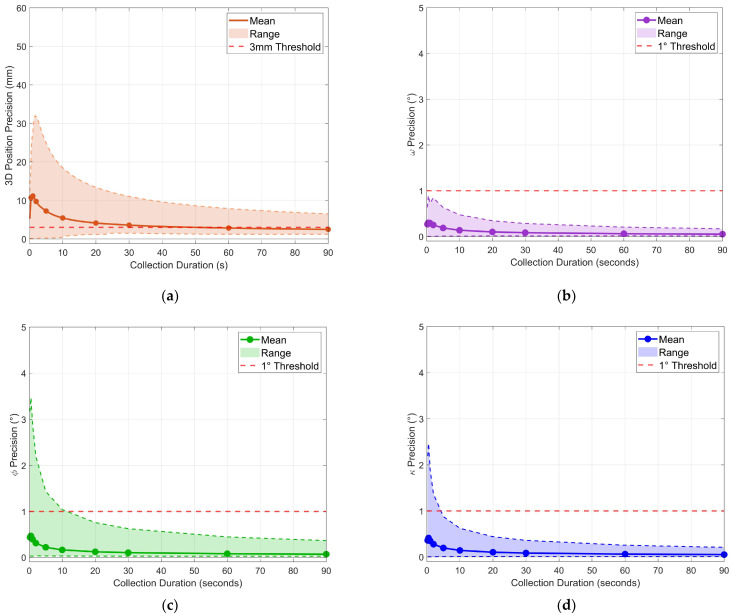
Running precision versus collection duration: (**a**) 3D position precision; (**b**) ω precision; (**c**) ϕ precision; (**d**) κ precision.

First-window analysis examined precision achievable from immediate data collection using windows extracted from the beginning of each stationary period. Figure 5 presents the mean 3D precision and individual Cardan angle precisions across all 30 grid points as a function of collection duration.

Mean position precision improved from 10.7 mm at 0.5 s to 3.6 mm at 30 s, demonstrating the expected inverse relationship between collection duration and measurement uncertainty. However, mean precision did not achieve the 3 mm threshold until between 45 s and 60 s. More critically, the maximum precision across all grid points remained above 6.5 mm, even after 90 s, revealing that some locations exhibited persistent high variability.

For the Cardan angles, ω precision exhibited the fastest convergence and tightest distribution, with maximum values below 0.8° for all durations. Precision for κ demonstrated rapid convergence, with maximum values falling below the 1° threshold by approximately 4–5 s. Precision for ϕ showed the slowest convergence among the three angles, with maximum values requiring approximately 11–12 s to fall below 1°.

#### 3.3.2. Sliding-Window Threshold Analysis

Sliding-window threshold analysis investigated whether allowing for an initial settling period could improve precision. Table 3 compares position precision achieved by immediate first windows versus windows positioned after threshold conditions were met.

The analysis demonstrated 100% success rates for all window durations up to 30 s, indicating that the 3 mm threshold could be achieved at all grid points given sufficient settling time. While first windows of 1 s achieved 11.1 mm mean position precision, the same 1 s window achieved 2.5 mm precision after a median wait time of only 0.60 s, confirming the presence of an initial settling period during which measurement precision stabilizes.

Among durations achieving 100% success, a clear trade-off emerged between wait time and collection duration. Shorter windows (0.5 s to 1 s) required median wait times of about 0.6 s but yielded median total collection times of 1.60 s or less. Longer windows (10 s to 30 s) required minimal median wait times but extended total collection times to 10 s or more. The maximum wait times revealed substantial spatial variability, with some grid locations requiring considerably longer settling periods than others.

Orientation measurements demonstrated fundamentally different convergence behavior. Table 4 presents corresponding sliding-window results for orientation precision, showing that all three Cardan angles achieved 100% success rates across all window durations, with median wait times of 0.00 s and maximum wait times of 0.30 s or less for durations up to 5 s. Near-immediate convergence confirmed that orientation estimates stabilized substantially faster than position estimates, establishing position as the critical bottleneck in data collection protocol optimization.

#### 3.3.3. Optimal Duration Recommendation

Based on this analysis, a 1 s duration was selected as optimal for the photogrammetric workflow. This duration achieved 100% success for position estimates, with a median total collection time of 1.60 s and a worst-case collection time of 2.50 s, while providing sufficient samples (20 epochs at 20 Hz) for robust precision estimation. Orientation measurements required no additional wait time at this duration, confirming that the 1 s window captured stable estimates for both position and orientation. Shorter windows, while faster, provided fewer samples for precision calculations.

To demonstrate the effectiveness of the optimized protocol, 95% confidence regions were computed for both immediate 1 s collection without settling and 1 s collection after threshold-based settling.

**Figure 6:**
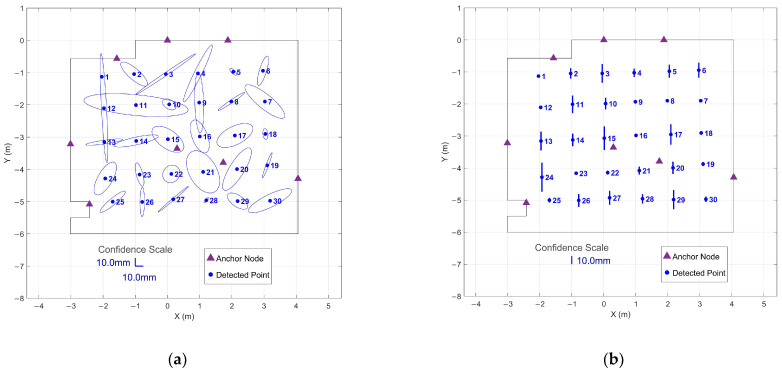
95% confidence regions from immediate 1 s data collection at all 30 grid points without settling period: (**a**) Horizontal confidence ellipses showing XY precision across the tracked area; (**b**) Vertical confidence intervals showing Z precision for each grid point.

Figure 6 shows confidence regions for immediate 1 s windows collected from the start of each stationary period. The horizontal confidence ellipses show highly variable and enlarged uncertainty regions, with a mean semi-major axis length of 27.0 mm and a maximum of 72.3 mm. Vertical confidence intervals similarly demonstrate poor precision, with a mean interval of 7.1 mm and a maximum of 19.5 mm. The elongated ellipses indicate strongly directional uncertainty patterns, suggesting that the system has not yet stabilized to provide uniform precision across measurement axes.

**Figure 7:**
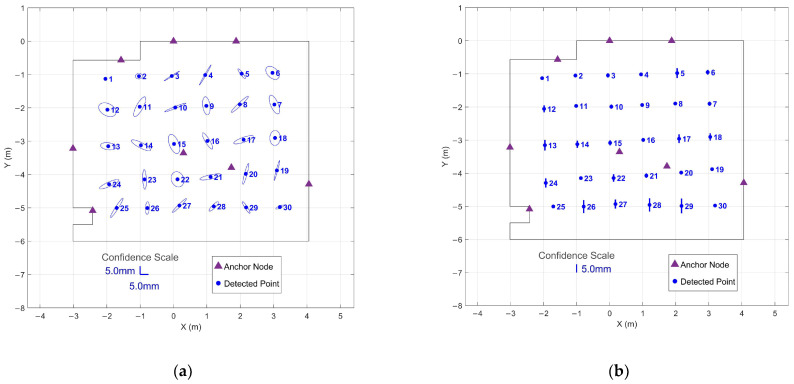
95% confidence regions from optimized 1 s data collection at all 30 grid points after threshold-based settling: (**a**) Horizontal confidence ellipses showing XY precision across the tracked area; (**b**) Vertical confidence intervals showing Z precision for each grid point.

The 95% confidence regions for the optimized approach using 1 s windows positioned after threshold-based settling are given in Figure 7. Horizontal confidence ellipses demonstrated a mean semi-major axis length of 5.3 mm and a maximum of 7.1 mm—comparable to the 90 s extended collection shown in Figure 4. The vertical confidence intervals averaged 1.7 mm with a maximum of 4.5 mm, nearly identical to the 90 s results (2.0 mm mean, 4.5 mm maximum). The dramatic reduction compared to Figure 6 demonstrates that the settling period addresses initialization noise rather than fundamental geometric limitations.

This comparison demonstrates that the initial settling period, rather than extended averaging, is the critical factor for achieving precise measurements. The optimized 1 s protocol captures steady-state performance while dramatically reducing collection time, enabling efficient large-scale surveys without compromising data quality.

### 3.4. Observation Uncertainty Quantification

Table 5 presents per-component precision statistics calculated from the 1 s threshold-qualified windows at each of the 30 grid points. For practical implementation in photogrammetric software, the RMS values across all grid points provide conservative yet realistic uncertainty estimates. For position, these uncertainties (1.6 mm in X, 1.7 mm in Y, and 1.1 mm in Z) represent the achievable precision using the optimized 1 s collection protocol with appropriate settling time and are consistent with the manufacturer’s 1.5 mm specification. For orientation, the uncertainties (0.08° for ω, 0.09° for ϕ, and 0.07° for κ) demonstrate sub-degree precision.

## 4. Discussion

This research characterized the measurement precision and temporal stability of the ZeroKey Quantum RTLS to establish optimal data collection protocols and determine whether its repeatability characteristics justify subsequent accuracy testing.

The automated stationary detection algorithm achieved 100% success for Profile 2 (Tooltip) across all observation durations, demonstrating that the envisioned walk-around workflow with brief pauses is technically feasible. Both the stationary detection and optimal duration analyses converge on the same conclusion: shorter data collection periods are superior for the Quantum RTLS. Just 1 s of data collection achieves both the 3 mm position precision threshold (after brief settling) and the 1° orientation precision threshold. The critical role of the settling period (median 0.6 s, maximum 1.5 s) rather than extended averaging indicates that operational workflows should allow the system to stabilize after operator motion ceases, after which measurements can be acquired rapidly.

The quantified per-component uncertainties provide the stochastic modeling parameters necessary for incorporating RTLS measurements as weighted constraints in photogrammetric bundle adjustment. The system’s millimeter-level position precision and sub-degree orientation precision demonstrate measurement repeatability appropriate for close-range photogrammetric applications. However, the relationship between these observation uncertainties and final reconstruction accuracy cannot be predicted without bundle adjustment testing.

This study characterized RTLS precision under a single anchor network configuration; sensitivity to anchor geometry warrants investigation in future work. Future work will also assess whether RTLS constraints yield accurate photogrammetric reconstructions, comparing results against traditional GCP-based approaches.

## Figures and Tables

**Table 1:** Stationary detection performance across five inertial measurement unit (IMU) profiles and three observation durations (30 s, 60 s, 90 s), showing number of detected periods, detection accuracy, undetected grid point IDs, and 3D precision statistics.

IMU Profile	Duration (s)	Number of Points Detected	Detection Rate (%)	Undetected Point IDs	Mean Precision (mm)	Minimum Precision (mm)	Maximum Precision (mm)
1	30	30	100%	-	2.9	0.7	9.0
	60	30	100%	-	2.8		
	90	27	90%	1, 22, 26	2.8		
2	30	30	100%	-	2.4	1.2	5.9
	60	30	100%	-	2.4		
	90	30	100%	-	2.4		
3	30	30	100%	-	3.3	1.0	15.5
	60	30	100%	-	3.2		
	90	30	100%	-	3.3		
4	30	30	100%	-	3.3	1.5	6.3
	60	29	90%	6	3.2		
	90	29	90%	6	3.2		
5	30	30	100%	-	4.1	1.1	12.1
	60	29	97%	6	4.0		
	90	28	93%	6, 28	4.0		

**Table 2:** Orientation statistics from 90 s stationary periods, showing spatial mean values (variation across 30 grid points) and temporal precision (mean standard deviation within each stationary period) for ω, ϕ, and κ Cardan angles.

Angle	Spatial Mean (°)	Minimum (°)	Maximum (°)	Temporal Precision (°)
ω	89.6 ± 0.6	88.1	90.6	0.07 ± 0.05
ϕ	−5.8 ± 8.3	−22.8	10.6	0.12 ± 0.09
κ	−0.4 ± 0.7	−1.5	1.2	0.08 ± 0.07

**Table 3:** Sliding-window position precision and wait time statistics for collection durations ranging from 0.25 s to 60 s.

Time (s)	Initial Precision (mm)	Success Rate (%)	Median Wait (s)	Maximum Wait (s)	Median Collection Time (s)	Maximum Collection Time (s)	Final Precision (s)
0.25	7.8	100%	0.38	1.35	0.63	1.60	2.6
0.50	10.7	100%	0.57	1.55	1.07	2.05	2.3
1	11.1	100%	0.60	1.50	1.60	2.50	2.5
2	9.7	100%	0.68	3.05	2.68	5.05	2.7
3	8.6	100%	0.60	3.00	3.60	6.00	2.7
5	7.3	100%	0.55	2.95	5.55	7.95	2.7
10	5.5	100%	0.45	2.80	10.45	12.80	2.7
20	4.1	100%	0.17	13.35	20.17	33.35	2.6
30	3.6	100%	0.03	5.45	30.03	35.45	2.6
45	3.1	97%	-				
60	2.8	97%	-				

**Table 4:** Sliding-window orientation precision and wait time statistics for collection durations ranging from 0.25 s to 60 s.

Time	Success Rate (%)	Median Wait (s)	Maximum Wait (s)	Median Collection Time (s)	Maximum Collection Time (s)
0.25	100%	0.00	0.25	0.25	0.50
0.50			0.30	0.50	0.80
1			0.15	1.00	1.15
2			0.15	2.00	2.15
3			0.20	3.00	3.20
5			0.15	5.00	5.15
10			0.05	10.00	10.05
20			0.00	20.00	20.00
30			0.00	30.00	30.00
45			0.00	45.00	45.00
60			0.00	60.00	60.00

**Table 5:** Per-component precision statistics for position and orientation measurements from optimized 1 s data collection across 30 grid points.

**Position (mm)**				
**Axis**	**Minimum**	**Maximum**	**Mean**	**RMS**
X	0.1	2.6	1.4	1.6
Y	0.1	2.6	1.6	1.7
Z	0.1	2.3	0.9	1.1
**Orientation (°)**				
**Angle**	**Minimum**	**Maximum**	**Mean**	**RMS**
ω	0.01	0.17	0.06	0.08
ϕ	0.02	0.32	0.06	0.09
κ	0.00	0.20	0.05	0.07

**Figure A1:**
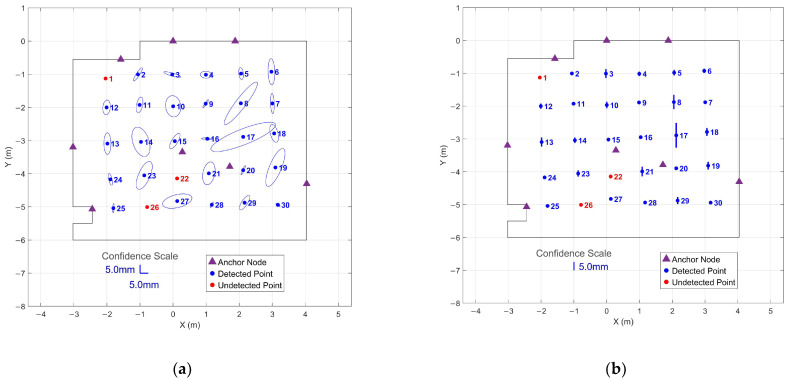
IMU Profile 1 95% confidence regions from 90 s stationary periods at all 30 grid points: (**a**) Horizontal confidence ellipses showing XY precision across the tracked area; (**b**) Vertical confidence intervals showing Z precision for each grid point.

**Figure A2:**
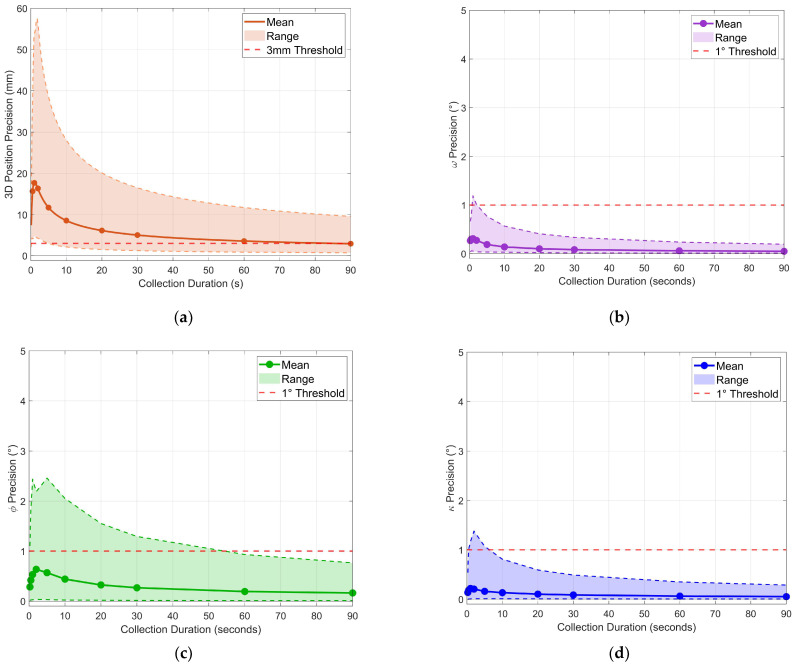
IMU Profile 1 running precision versus collection duration: (**a**) 3D position precision; (**b**) ω precision; (**c**) ϕ precision; (**d**) κ precision.

**Figure A3:**
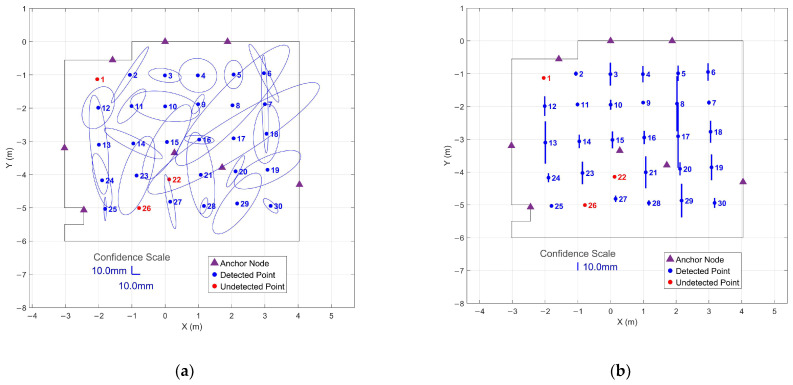
IMU Profile 1 95% confidence regions from immediate 1 s data collection at all 30 grid points without settling period: (**a**) Horizontal confidence ellipses showing XY precision across the tracked area; (**b**) Vertical confidence intervals showing Z precision for each grid point.

**Figure A4:**
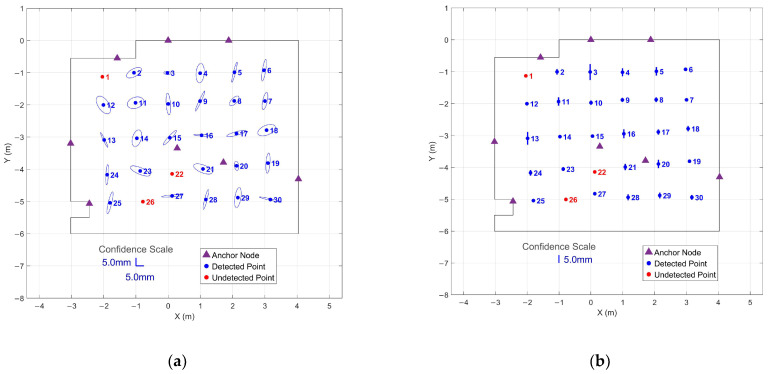
IMU Profile 1 95% confidence regions from optimized 1 s data collection at all 30 grid points after threshold-based settling: (**a**) Horizontal confidence ellipses showing XY precision across the tracked area; (**b**) Vertical confidence intervals showing Z precision for each grid point.

**Figure A5:**
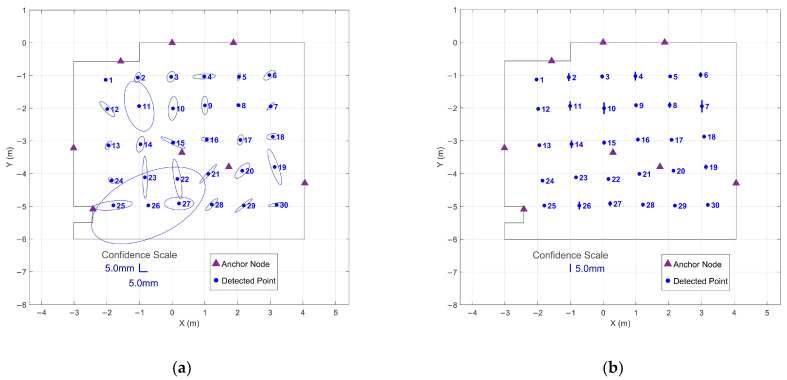
IMU Profile 3 95% confidence regions from 90 s stationary periods at all 30 grid points: (**a**) Horizontal confidence ellipses showing XY precision across the tracked area; (**b**) Vertical confidence intervals showing Z precision for each grid point.

**Figure A6:**
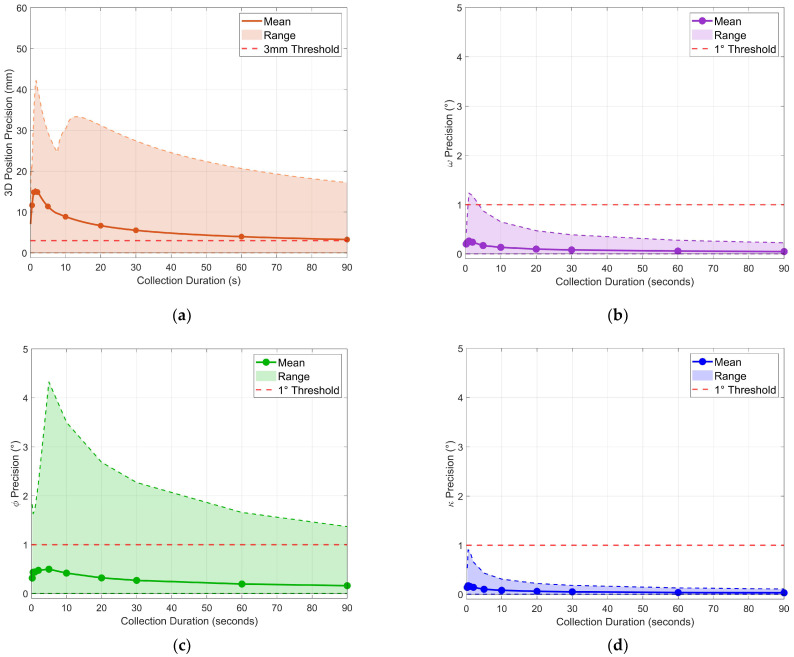
IMU Profile 3 running precision versus collection duration: (**a**) 3D position precision; (**b**) ω precision; (**c**) ϕ precision; (**d**) κ precision.

**Figure A7:**
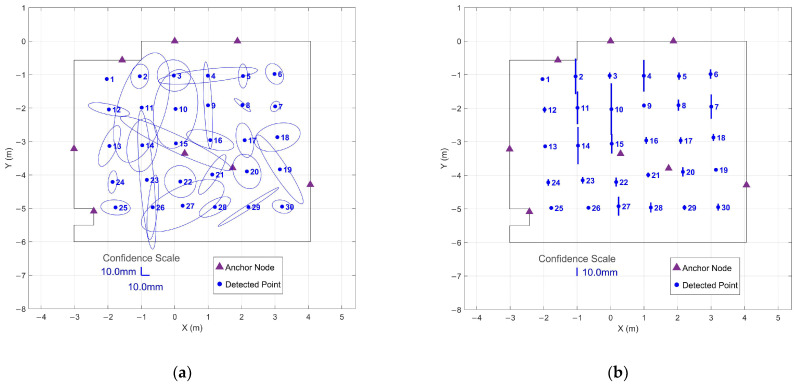
IMU Profile 3 95% confidence regions from immediate 1 s data collection at all 30 grid points without settling period: (**a**) Horizontal confidence ellipses showing XY precision across the tracked area; (**b**) Vertical confidence intervals showing Z precision for each grid point.

**Figure A8:**
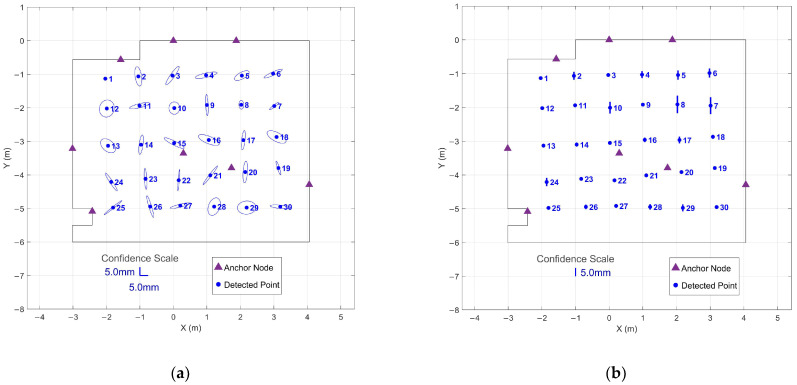
IMU Profile 3 95% confidence regions from optimized 1 s data collection at all 30 grid points after threshold-based settling: (**a**) Horizontal confidence ellipses showing XY precision across the tracked area; (**b**) Vertical confidence intervals showing Z precision for each grid point.

**Figure A9:**
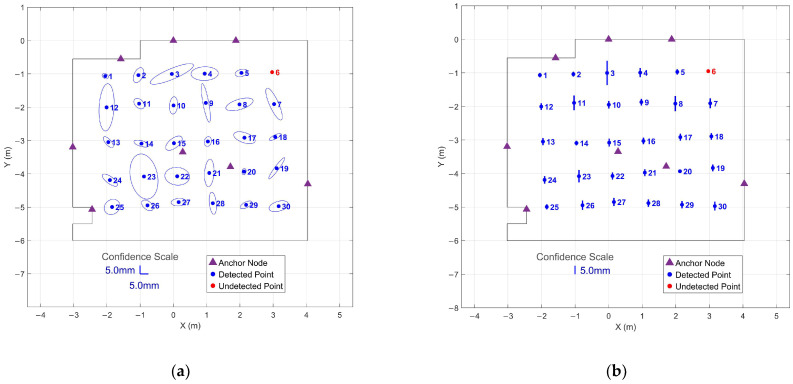
IMU Profile 4 95% confidence regions from 90 s stationary periods at all 30 grid points: (**a**) Horizontal confidence ellipses showing XY precision across the tracked area; (**b**) Vertical confidence intervals showing Z precision for each grid point.

**Figure A10:**
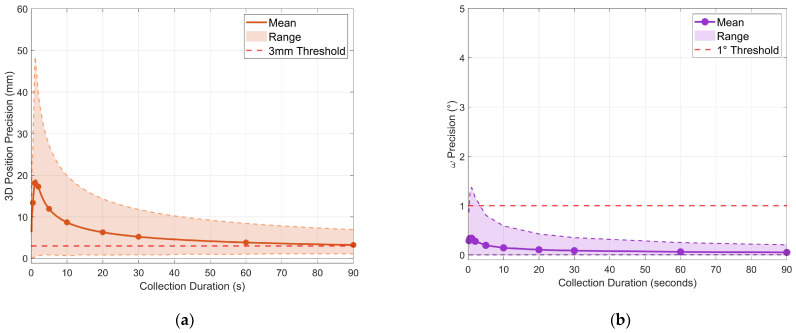

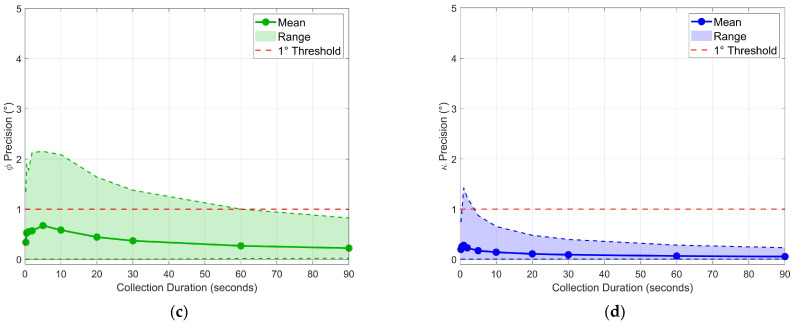
IMU Profile 4 running precision versus collection duration: (**a**) 3D position precision; (**b**) ω precision; (**c**) ϕ precision; (**d**) κ precision.

**Figure A11:**
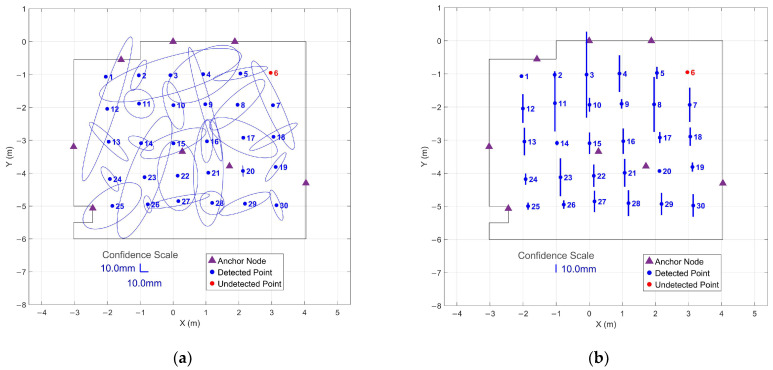
IMU Profile 4 95% confidence regions from immediate 1 s data collection at all 30 grid points without settling period: (**a**) Horizontal confidence ellipses showing XY precision across the tracked area; (**b**) Vertical confidence intervals showing Z precision for each grid point.

**Figure A12:**
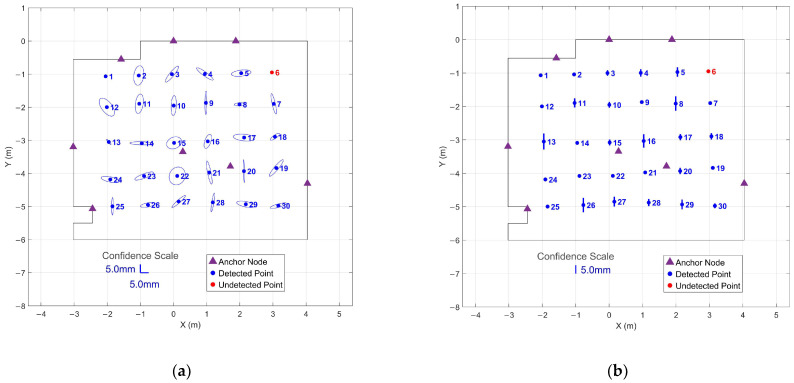
IMU Profile 4 95% confidence regions from optimized 1 s data collection at all 30 grid points after threshold-based settling: (**a**) Horizontal confidence ellipses showing XY precision across the tracked area; (**b**) Vertical confidence intervals showing Z precision for each grid point.

**Figure A13:**
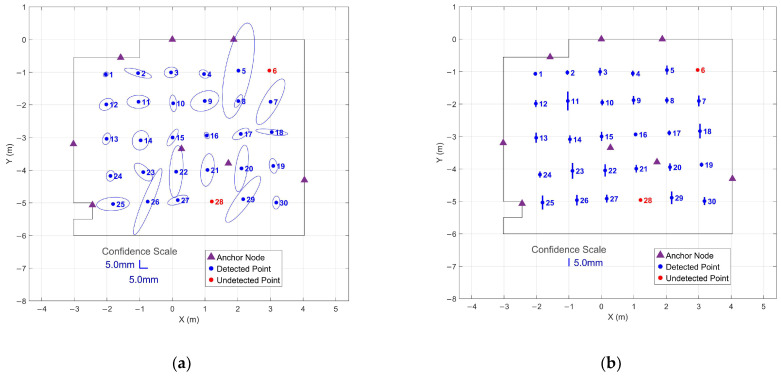
IMU Profile 5 95% confidence regions from 90 s stationary periods at all 30 grid points: (**a**) Horizontal confidence ellipses showing XY precision across the tracked area; (**b**) Vertical confidence intervals showing Z precision for each grid point.

**Figure A14:**
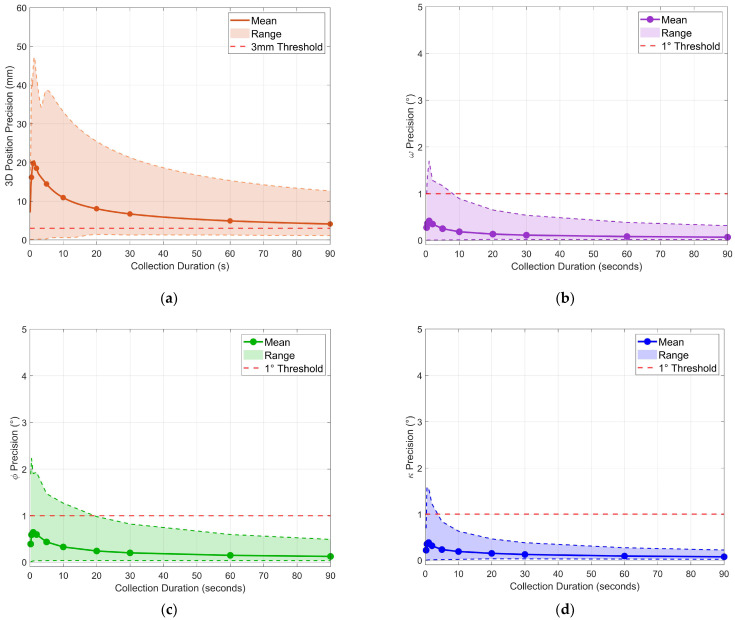
IMU Profile 5 running precision versus collection duration: (**a**) 3D position precision; (**b**) ω precision; (**c**) ϕ precision; (**d**) κ precision.

**Figure A15:**
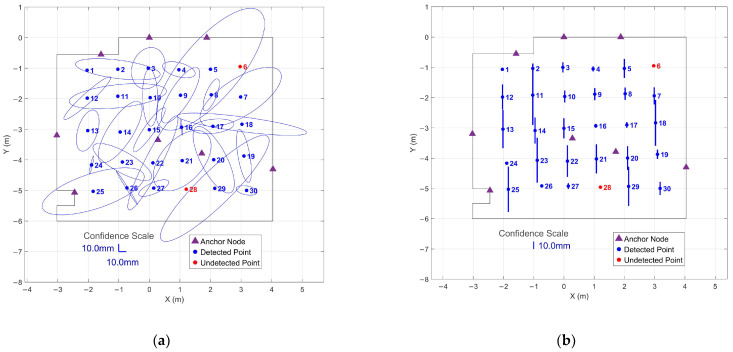
IMU Profile 5 95% confidence regions from immediate 1 s data collection at all 30 grid points without settling period: (**a**) Horizontal confidence ellipses showing XY precision across the tracked area; (**b**) Vertical confidence intervals showing Z precision for each grid point.

**Figure A16:**
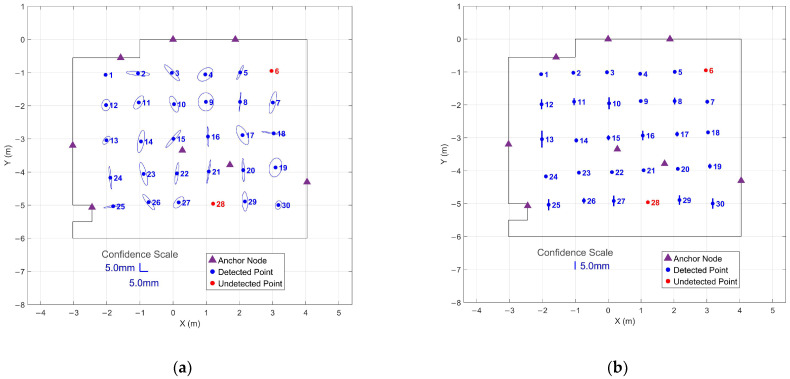
IMU Profile 5 95% confidence regions from optimized 1 s data collection at all 30 grid points after threshold-based settling: (**a**) Horizontal confidence ellipses showing XY precision across the tracked area; (**b**) Vertical confidence intervals showing Z precision for each grid point.

## Data Availability

The data presented in this study can be made available upon request from the corresponding author.

## Abbreviations

The following abbreviations are used in this manuscript:

BLE	Bluetooth Low Energy
GCP	ground control point
GNSS	global navigation satellite system
IMU	inertial measurement unit
IPS	indoor positioning system
ISO	integrated sensor orientation
RMS	root mean square
RTLS	real-time location system
ToF	time-of-flight
UAV	unmanned aerial vehicle
UWB	ultra-wideband

## Appendix A

### Appendix A.1. IMU Profile 1 Results

**Table A1:** IMU Profile 1 orientation statistics from 90 s stationary periods, showing spatial mean values (variation across 30 grid points) and temporal precision (mean standard deviation within each stationary period) for ω, ϕ, and κ Cardan angles.

Angle	Spatial Mean (°)	Minimum (°)	Maximum (°)	Temporal Precision (°)
ω	92.0 ± 0.3	91.3	92.4	0.05 ± 0.05
ϕ	−5.1 ± 4.3	−11.9	6.1	0.16 ± 0.15
κ	−0.2 ± 0.5	−0.9	1.0	0.05 ± 0.07

**Table A2:** IMU Profile 1 sliding-window position precision and wait time statistics for collection durations ranging from 0.25 s to 60 s.

Time (s)	Initial Precision (mm)	Success Rate (%)	Median Wait (s)	Maximum Wait (s)	Median Collection Time (s)	Maximum Collection Time (s)	Final Precision (s)
0.25	11.2	100%	0.65	2.25	0.90	2.50	2.4
0.50	15.7	100%	0.90	2.25	1.40	2.75	2.5
1	17.7	100%	1.15	2.45	2.15	3.45	2.7
2	16.4	100%	1.10	2.35	3.10	4.35	2.8
3	14.4	100%	1.00	2.30	4.00	5.30	2.8
5	11.7	100%	0.90	2.05	5.90	7.05	2.8
10	8.6	100%	0.75	1.95	10.75	11.95	2.8
20	6.1	100%	0.55	1.85	20.55	21.85	2.8
30	5.0	100%	0.30	1.65	30.30	31.65	2.7
45	4.1	100%	0.00	1.25	45.00	46.25	2.6
60	3.6	100%	0.00	0.70	60.00	60.70	2.5

**Table A3:** IMU Profile 1 sliding-window orientation precision and wait time statistics for collection durations ranging from 0.25 s to 60 s.

Time	Success Rate (%)	Median Wait (s)	Maximum Wait (s)	Median Collection Time (s)	Maximum Collection Time (s)
0.25	100%	0.00	0.10	0.25	0.35
0.50			0.45	0.50	0.95
1			0.40	1.00	1.40
2			1.85	2.00	3.85
3			1.80	3.00	4.80
5			1.75	5.00	6.75
10			1.60	10.00	11.60
20			1.30	20.00	21.30
30			1.00	30.00	31.00
45			0.60	45.00	45.60
60			0.00	60.00	60.00

**Table A4:** IMU Profile 1 per-component precision statistics for position and orientation measurements from optimized 1 s data collection across 30 grid points.

**Position (mm)**				
**Axis**	**Minimum**	**Maximum**	**Mean**	**RMS**
X	0.3	2.8	1.3	1.5
Y	0.2	2.8	1.8	2.0
Z	0.2	2.6	0.9	1.0
**Orientation (°)**				
**Angle**	**Minimum**	**Maximum**	**Mean**	**RMS**
ω	0.01	0.17	0.05	0.07
ϕ	0.01	0.13	0.06	0.06
κ	0.00	0.03	0.01	0.02

### Appendix A.2. IMU Profile 3 Results

**Table A5:** IMU Profile 3 orientation statistics from 90 s stationary periods, showing spatial mean values (variation across 30 grid points) and temporal precision (mean standard deviation within each stationary period) for ω, ϕ, and κ Cardan angles.

Angle	Spatial Mean (°)	Minimum (°)	Maximum (°)	Temporal Precision (°)
ω	90.8 ± 0.6	89.8	91.7	0.04 ± 0.07
ϕ	−4.7 ± 6.9	−16.9	13.9	0.16 ± 0.23
κ	−0.1 ± 0.5	−1.2	1.0	0.02 ± 0.04

**Table A6:** IMU Profile 3 sliding-window position precision and wait time statistics for collection durations ranging from 0.25 s to 60 s.

Time (s)	Initial Precision (mm)	Success Rate (%)	Median Wait (s)	Maximum Wait (s)	Median Collection Time (s)	Maximum Collection Time (s)	Final Precision (s)
0.25	9.6	100%	0.47	1.80	0.72	2.05	2.4
0.50	11.7	100%	0.75	2.40	1.25	2.90	2.6
1	14.8	100%	1.02	3.40	2.02	4.40	2.6
2	14.9	100%	1.05	3.40	3.05	5.40	2.6
3	13.7	100%	1.02	3.40	4.02	6.40	2.7
5	11.4	100%	0.97	10.65	5.97	15.65	2.7
10	8.9	100%	0.80	10.45	10.80	20.45	2.7
20	6.7	100%	0.53	9.95	20.53	29.95	2.7
30	5.5	100%	0.35	9.85	30.35	39.85	2.6
45	4.6	100%	0.20	9.75	45.20	54.75	2.5
60	4.0	100%	0.05	9.65	60.05	69.65	2.5

**Table A7:** IMU Profile 3 sliding-window orientation precision and wait time statistics for collection durations ranging from 0.25 s to 60 s.

Time	Success Rate (%)	Median Wait (s)	Maximum Wait (s)	Median Collection Time (s)	Maximum Collection Time (s)
0.25	100%	0.00	0.10	0.25	0.35
0.50			0.30	0.50	0.80
1			0.30	1.00	1.30
2			1.00	2.00	3.00
3			2.60	3.00	5.60
5			6.30	5.00	11.30
10			6.15	10.00	16.15
20			5.95	20.00	25.95
30			5.75	30.00	35.75
45			5.40	45.00	50.40
60			5.05	60.00	65.05

**Table A8:** IMU Profile 3 per-component precision statistics for position and orientation measurements from optimized 1 s data collection across 30 grid points.

**Position (mm)**				
**Axis**	**Minimum**	**Maximum**	**Mean**	**RMS**
X	0.0	2.7	1.5	1.7
Y	0.0	2.6	1.7	1.8
Z	0.0	2.6	0.8	1.0
**Orientation (°)**				
**Angle**	**Minimum**	**Maximum**	**Mean**	**RMS**
ω	0.00	0.10	0.03	0.04
ϕ	0.00	0.08	0.03	0.03
κ	0.00	0.05	0.01	0.02

### Appendix A.3. IMU Profile 4 Results

**Table A9:** IMU Profile 4 orientation statistics from 90 s stationary periods, showing spatial mean values (variation across 30 grid points) and temporal precision (mean standard deviation within each stationary period) for ω, ϕ, and κ Cardan angles.

Angle	Spatial Mean (°)	Minimum (°)	Maximum (°)	Temporal Precision (°)
ω	90.8 ± 0.9	88.8	92.1	0.04 ± 0.06
ϕ	−6.5 ± 5.0	−14.2	2.5	0.23 ± 0.16
κ	−0.6 ± 0.6	−1.5	0.3	0.04 ± 0.06

**Table A10:** IMU Profile 4 sliding-window position precision and wait time statistics for collection durations ranging from 0.25 s to 60 s.

Time (s)	Initial Precision (mm)	Success Rate (%)	Median Wait (s)	Maximum Wait (s)	Median Collection Time (s)	Maximum Collection Time (s)	Final Precision (s)
0.25	10.9	100%	0.55	1.60	0.80	1.85	2.4
0.50	13.4	100%	1.00	2.70	1.50	3.20	2.5
1	18.1	100%	1.15	2.70	2.15	3.70	2.6
2	17.3	100%	1.15	2.65	3.15	4.65	2.7
3	4.9	100%	1.10	2.60	4.10	5.60	2.7
5	12.0	100%	1.10	2.40	6.10	7.40	2.8
10	8.7	100%	1.00	2.00	11.00	12.00	2.7
20	6.3	100%	0.75	1.90	20.75	21.90	2.7
30	5.2	100%	0.50	1.75	30.50	31.75	2.8
45	4.4	100%	0.35	1.45	45.35	46.45	2.7
60	3.8	100%	0.20	1.25	60.20	61.25	2.7

**Table A11:** IMU Profile 4 sliding-window orientation precision and wait time statistics for collection durations ranging from 0.25 s to 60 s.

Time	Success Rate (%)	Median Wait (s)	Maximum Wait (s)	Median Collection Time (s)	Maximum Collection Time (s)
0.25	100%	0.00	0.15	0.25	0.40
0.50			0.75	0.50	1.25
1			0.75	1.00	1.75
2			1.35	2.00	3.35
3			1.30	3.00	4.30
5			1.70	5.00	6.70
10			1.40	10.00	10.40
20			0.75	20.00	20.75
30			0.55	30.00	30.55
45			0.20	45.00	45.20
60			0.05	60.00	60.05

**Table A12:** IMU Profile 4 per-component precision statistics for position and orientation measurements from optimized 1 s data collection across 30 grid points.

**Position (mm)**				
**Axis**	**Minimum**	**Maximum**	**Mean**	**RMS**
X	0.1	2.9	1.4	1.6
Y	0.2	2.9	1.6	1.8
Z	0.1	2.4	0.9	1.1
**Orientation (°)**				
**Angle**	**Minimum**	**Maximum**	**Mean**	**RMS**
ω	0.00	0.06	0.02	0.03
ϕ	0.00	0.26	0.11	0.13
κ	0.00	0.08	0.02	0.02

### Appendix A.4. IMU Profile 5 Results

**Table A13:** IMU Profile 5 orientation statistics from 90 s stationary periods, showing spatial mean values (variation across 30 grid points) and temporal precision (mean standard deviation within each stationary period) for ω, ϕ, and κ Cardan angles.

Angle	Spatial Mean (°)	Minimum (°)	Maximum (°)	Temporal Precision (°)
ω	90.2 ± 0.7	88.4	91.4	0.05 ± 0.08
ϕ	−6.6 ± 5.3	−19.5	3.5	0.12 ± 0.11
κ	0.1 ± 0.7	−0.7	2.1	0.06 ± 0.06

**Table A14:** IMU Profile 5 sliding-window position precision and wait time statistics for collection durations ranging from 0.25 s to 60 s.

Time (s)	Initial Precision (mm)	Success Rate (%)	Median Wait (s)	Maximum Wait (s)	Median Collection Time (s)	Maximum Collection Time (s)	Final Precision (s)
0.25	12.2	100%	0.78	1.90	1.03	2.15	2.4
0.50	16.2	100%	1.05	2.65	1.55	3.15	2.4
1	19.8	100%	1.15	2.60	2.15	3.60	2.7
2	18.5	100%	1.12	3.70	3.12	5.70	2.7
3	16.6	100%	1.10	3.70	4.10	6.70	2.7
5	14.5	100%	1.08	3.65	6.08	8.65	2.7
10	10.9	100%	0.95	3.55	10.95	13.55	2.8
20	8.1	100%	0.75	3.40	20.75	23.40	2.8
30	6.7	100%	0.57	3.35	30.57	33.35	2.8
45	5.6	100%	0.42	3.30	45.42	48.30	2.7
60	4.9	100%	0.10	3.10	60.10	63.10	2.6

**Table A15:** IMU Profile 5 sliding-window orientation precision and wait time statistics for collection durations ranging from 0.25 s to 60 s.

Time	Success Rate (%)	Median Wait (s)	Maximum Wait (s)	Median Collection Time (s)	Maximum Collection Time (s)
0.25	100%	0.00	0.20	0.25	0.45
0.50			0.30	0.50	0.80
1			0.80	1.00	1.80
2			0.75	2.00	2.75
3			0.70	3.00	3.70
5			2.55	5.00	7.55
10			1.90	10.00	11.90
20			0.00	20.00	20.00
30			0.00	30.00	30.00
45			0.00	45.00	45.00
60			0.00	60.00	60.00

**Table A16:** IMU Profile 5 per-component precision statistics for position and orientation measurements from optimized 1 s data collection across 30 grid points.

**Position (mm)**				
**Axis**	**Minimum**	**Maximum**	**Mean**	**RMS**
X	0.1	3.0	1.2	1.4
Y	0.1	2.9	1.9	2.1
Z	0.1	2.6	0.9	1.1
**Orientation (°)**				
**Angle**	**Minimum**	**Maximum**	**Mean**	**RMS**
ω	0.00	0.19	0.07	0.09
ϕ	0.02	0.52	0.07	0.12
κ	0.00	0.05	0.02	0.02
